# Erythropoietin Signaling Regulates Key Epigenetic and Transcription Networks in Fetal Neural Progenitor Cells

**DOI:** 10.1038/s41598-017-14366-0

**Published:** 2017-10-30

**Authors:** Christina Sollinger, Jacquelyn Lillis, Jeffrey Malik, Michael Getman, Chris Proschel, Laurie Steiner

**Affiliations:** 1 Department of Pediatrics, University of Rochester, Rochester, New York, USA; 2 Functional Genomic Center, University of Rochester, Rochester, New York, USA; 3 Department of Biomedical Genetics, University of Rochester, Rochester, New York, USA

## Abstract

Erythropoietin (EPO) and its receptor are highly expressed in the developing nervous system, and exogenous EPO therapy is potentially neuroprotective, however the epigenetic and transcriptional changes downstream of EPO signaling in neural cells are not well understood. To delineate epigenetic changes associated with EPO signaling, we compared histone H3 lysine 4 dimethylation (H3K4me2) in EPO treated and control fetal neural progenitor cells, identifying 1,150 differentially bound regions. These regions were highly enriched near protein coding genes and had significant overlap with H4Acetylation, a mark of active regulatory elements. Motif analyses and co-occupancy studies revealed a complex regulatory network underlying the differentially bound regions, including previously identified mediators of EPO signaling (STAT5, STAT3), and novel factors such as REST, an epigenetic modifier central to neural differentiation and plasticity, and NRF1, a key regulator of antioxidant response and mitochondrial biogenesis. Global transcriptome analyses on neural tubes isolated from E9.0 EpoR-null and littermate control embryos validated our *in vitro* findings, further suggesting a role for REST and NRF1 downstream of EPO signaling. These data support a role for EPO in regulating the survival, proliferation, and differentiation of neural progenitor cells, and suggest a basis for its function in neural development and neuroprotection.

## Introduction

Erythropoietin (EPO) is a glycoprotein hormone that is best known for its essential role in the proliferation, maturation, and survival of erythroid progenitor cells^1,2^. EPO and its receptor, EpoR, are also highly expressed in the developing nervous system of both humans and mice^3–8^. EpoR is expressed on many types of cells in the nervous system including mature neurons, astrocytes, glial cells and CNS capillary pericytes^6–9^. EpoR is also highly expressed on neural progenitor cells, and EPO treatment of neural progenitor cells promotes neurogenesis both *in vitro* and *in vivo* ^10^. EpoR-null embryos develop neurologic abnormalities prior to the development of lethal anemia, including decreased numbers of neural progenitor cells and hypoplasia of the forebrain and neural epithelium^2,3,5^. Endogenous EPO signaling is required for the normal proliferation of neural progenitor cells^4^, and neural progenitor cells isolated from EpoR-null embryos have higher rates of apoptosis and increased sensitivity to hypoxia compared to controls, even following selective restoration of EpoR in erythroid progenitors^3–5^. Together, these data suggest that EPO signaling regulates the survival, proliferation, and specification of neural progenitor cells, however the molecular mechanisms that promote these functions are not well understood.

Exogenous EPO has been extensively studied as a potential neuroprotective agent in several disease states that impact neonates. Exogenous EPO therapy has shown particular promise as a neuroprotective agent in neonates affected by Hypoxic Ischemic Encephalopathy (HIE), a hypoxic brain insult that occurs at or around the time of birth and is associated with significant neurodevelopmental morbidity^11–17^. Exogenous EPO therapy is also being investigated as a neuroprotective agent to improve neurodevelopmental outcomes for preterm infants^18,19^ and animal studies suggest that exogenous EPO therapy may be neuroprotective for infants impacted by neonatal stroke^20^. The neuroprotective effects of EPO treatment are likely due to its action on multiple cell types. Data from animal models suggest that in the setting of hypoxic or ischemic insult exogenous EPO therapy decreases apoptosis, promotes neurogenesis, enhances oligodendrocyte development, and promotes revascularization^21–24^. Gene expression analyses performed on sections of brain in murine models of hypoxic-ischemic damage demonstrate that EPO treatment promotes the expression of anti-apoptotic genes such as BCL2 and BLCXL, and suppresses the expression of pro-apoptotic genes such as BAX and BIM^14,25^. Data regarding the gene expression changes following EPO treatment in specific populations of neural cells is lacking and the mechanisms by which EPO promotes neurogenesis are not well understood.

The most comprehensive studies on the molecular mechanisms downstream of EPO signaling have been done in the context of erythropoiesis. In definitive erythroid progenitors EpoR activation results in phosphorylation of JAK2 and activation of downstream effectors, including STAT5 and AKT, that translocate to the nucleus where they interact with erythroid-specific transcription factors to drive transcriptional changes that promote the proliferation, maturation, and survival of erythroid progenitors^26–28^. In non-erythroid cells, the molecular events involved in EPO signaling are not as well characterized. EPO signaling in neural cells is thought to involve heterodimerization with the common beta chain receptor^29,30^, which is dispensable for erythropoiesis but necessary for the neuroprotective effects of exogenous EPO therapy^31–33^. The molecular events downstream of EPO signaling in neural cells are also complex, with STAT5, STAT3, phosphatidylinositol 3-kinase (PI3-k), NF-kβ, and GATA3 all reported to be downstream of EpoR activation^34–37^.

The majority of studies on EPO signaling in the nervous system have been conducted in tumor-derived cell lines or models of hypoxic-ischemic insult in adult animals that do not accurately recapitulate the developing nervous system. As a result, the molecular mechanisms that underlie the function of EPO in fetal neural progenitor cells and their differentiated progeny are largely unknown. We hypothesized that identification of the epigenomic and transcriptomic changes downstream of EPO signaling in fetal neural progenitor cells would provide valuable insights into the molecular mechanisms underlying the function of EPO signaling in the developing nervous system. To that end, we sought to delineate the epigenetic and transcriptomic changes downstream of EPO signaling in human fetal neural progenitor cells.

## Results

### EPO treatment alters the epigenetic landscape of human neural progenitor cells

**Figure 1:**
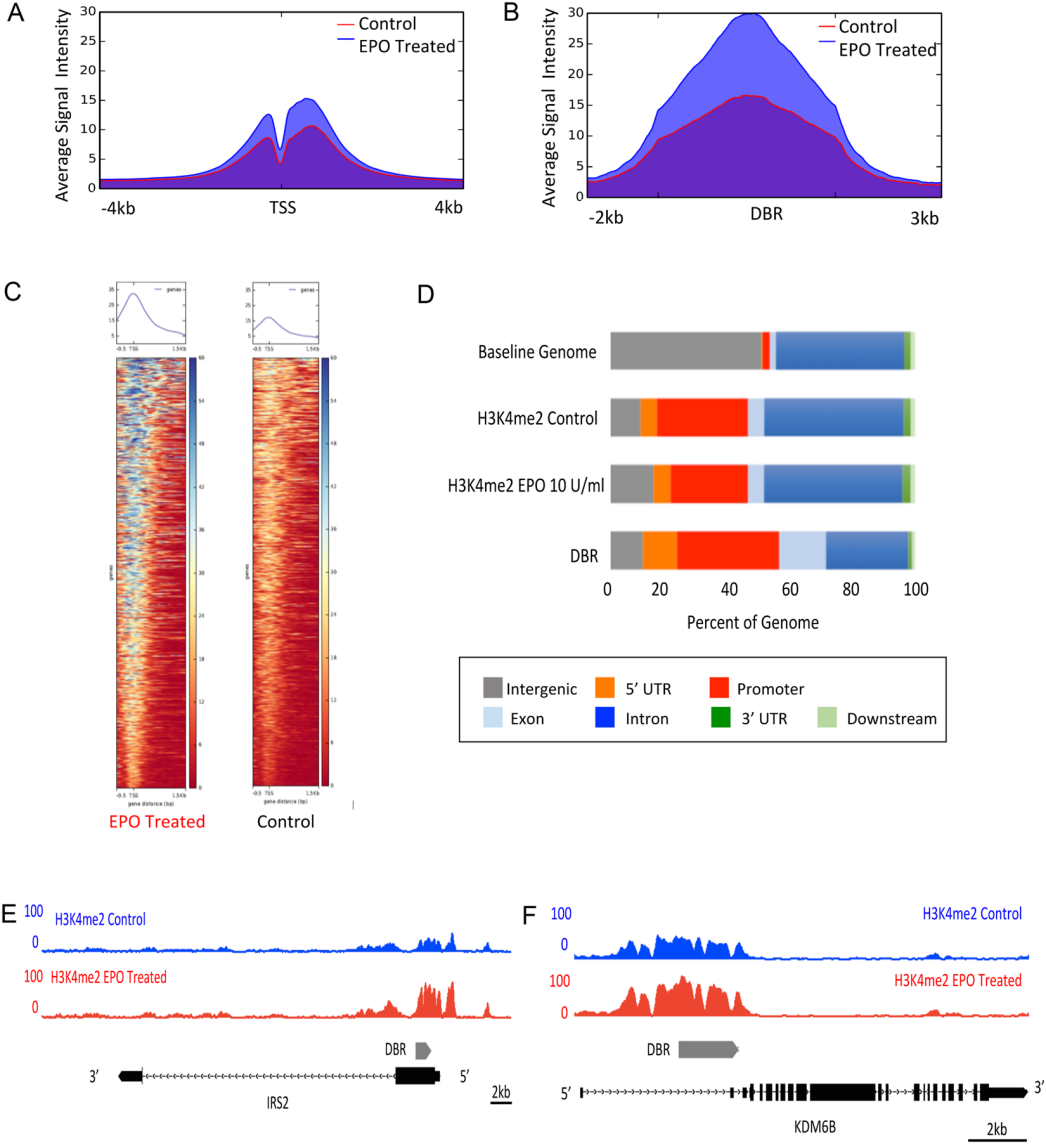
EPO treatment is associated with changes in the epigenetic landscape of human neural progenitor cells. (**A**) Average signal intensity for H3K4me2 over transcription start sites (TSS) in EPO treated and control samples. (**B**) Average signal intensity for H3K4me2 for the EPO treated and control samples at the differentially bound regions (DBR). (**C**) Heat maps representing the level of H3K4me2 occupancy at the 1150 DBR in the EPO treated and control samples. (**D**) Relationship of the DBR to known genomic features. (**E**) Example of H3K4me2 occupancy in the EPO treated and control samples at the IRS2 locus. Grey bar represents DBR. (**F**) Example of H3K4me2 occupancy in the EPO treated and control samples at the KDM6B locus. Grey bar represents DBR.

We used chromatin immunoprecipitation coupled with high throughput sequencing (ChIP-seq) to profile changes in the epigenetic landscape associated with EPO treatment in human fetal myc-immortalized neural progenitor cells initially isolated from 10-week midbrain. ChIP was done using an antibody for histone H3, lysine 4 dimethylation (H3K4me2), which marks active and poised enhancers and transcription start sites^38^. ChIP assays were done following 24 hours of treatment with EPO (10U/ml) and in untreated control cultures. The EPO concentration of 10U/ml was chosen because it approximates the serum EPO concentration of infants treated with exogenous EPO therapy for neuroprotection^15,39^. Each ChIP-seq experiment was done in duplicate. There was a high correlation between experimental replicates (Fig. S1) and the number of uniquely mapped reads was similar between samples (Table S1). Peaks were called using MACS^40^ with default parameters, with a p-value < 0.05 considered significant. The EPO treated samples had a higher overall number of statistically significant H3K4me2 peaks than control samples (33,087 and 24,197, respectively) as well as a modest increase in H3K4me2 signal over transcription start sites (Fig. 1A). As expected, the regions of H3K4me2 occupancy for both the EPO treated and control cells were generally located in close proximity to protein coding genes and enriched near transcription start sites (Fig. 1D).

Accurately identifying regions of enrichment in ChIP-seq data sets where the target of interest occupies broad regions of DNA, such as histone modifications, can be challenging because most peak calling algorithms are designed to identify narrow peaks of factor occupancy^41^. We therefore used DiffReps, an algorithm specifically designed to identify differential histone occupancy between two conditions^41^, to identify significant regions of differential H3K4me2 occupancy between the EPO treated and control samples. Using DiffReps, 1150 differentially bound regions (DBR) were identified (p < 0.001; Fig. 1B–F). We validated a subset of those regions using quantitative ChIP (Fig. S2). At the majority of the DBR, there was increased H3K4me2 occupancy in the EPO treated samples compared to control, the magnitude of which was significantly greater than the modest increase in H3K4me2 occupancy associated with EPO treatment at TSS (Fig. 1A,B). Consistent with a role in transcriptional regulation, the majority of DBR were located near protein coding genes, and were highly enriched at promoter regions (p < 10–322, Fig. 1D). These results suggest that the EPO signaling promotes changes in the epigenetic landscape of neural progenitor cells at regulatory elements, such as promoters, that are likely to facilitate changes in gene expression.

### EPO treatment promotes gene expression changes associated with neural development and neural protection

**Figure 2:**
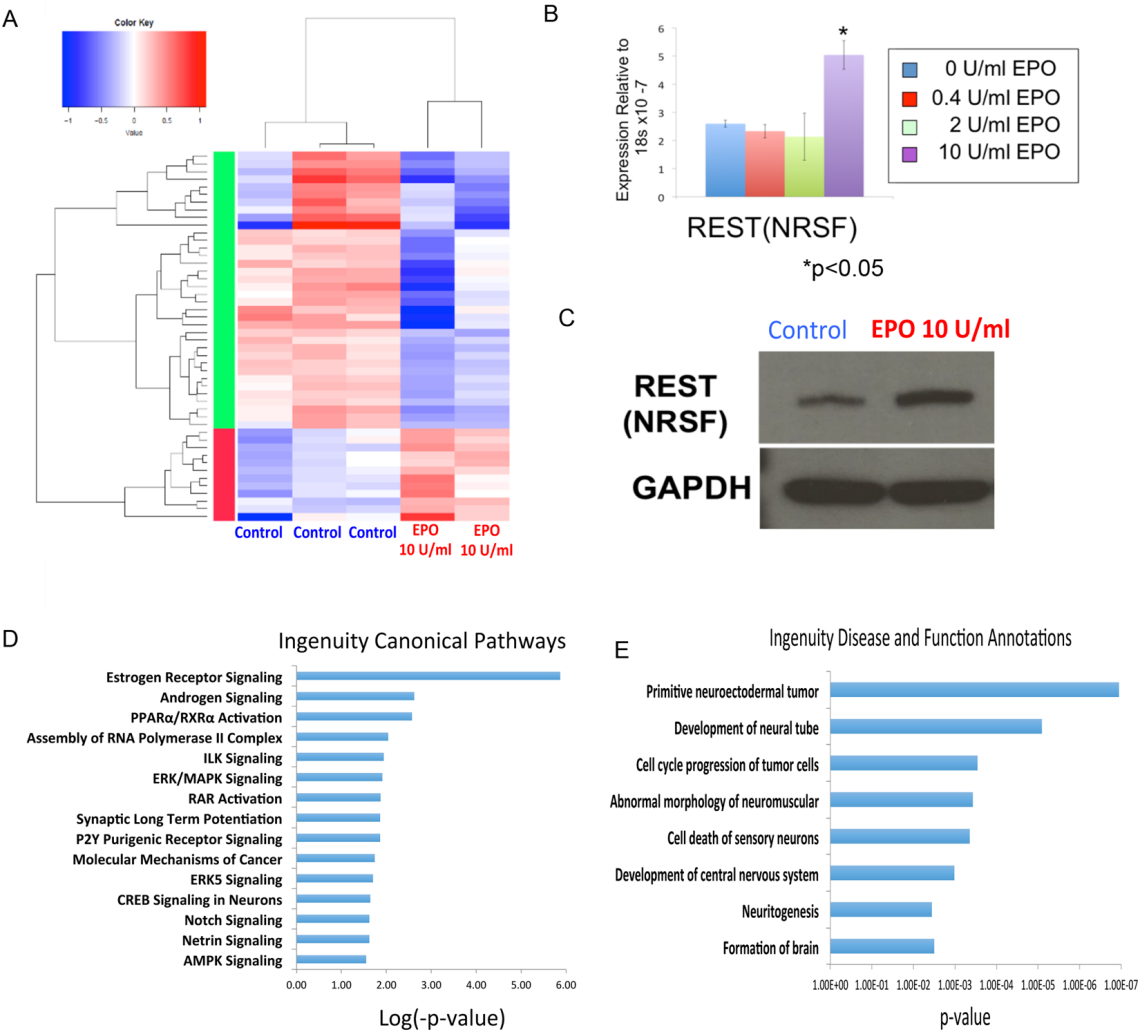
Gene expression changes associated with EPO treatment in neural progenitor cells. (**A**) Heat map of genes differentially expressed in EPO treated and control samples. (**B**) Quantitative PCR demonstrating REST mRNA expression in control cells and in cells treated with EPO concentrations ranging from 0.4U/ml to 10U/ml. (**C**) REST protein levels in control and EPO treated (10U/ml) cells. The full image of the blot is in Fig S7. (**D**) Canonical pathways by identified by Ingenuity Pathway Analyses of the differentially expressed genes. (**E**) Disease and Function annotations identified by Ingenuity Pathway Analyses of the differentially expressed genes.

To determine the gene expression changes downstream of EPO-signaling, global transcriptome analyses were done in human fetal neural progenitor cells following 24 hours of treatment with EPO (10 U/ml) and in untreated control cells. The RNA-seq studies were done in duplicate, and the replicates were highly correlated. (Fig. S3) As expected following the addition of a single cytokine in steady-state culture conditions, the changes in gene expression were modest. In total, 566 genes were differentially expressed (p value < 0.005, FDR < 0.05; Fig. 2A). 393 genes had higher expression in the EPO treated samples and 173 genes had higher expression in the control samples. One of the most interesting differentially expressed genes was REST (RE1 Silencing Transcription Factor), a master regulator of neurogenesis^42,43^. We validated the change in REST expression at both the RNA (Fig. 2B) and protein level (Fig. 2C) following treatment with 10U/ml of EPO, the dose used for the RNA-seq studies. We also assessed REST expression following treatment with lower doses of EPO (0.4–2 U/ml), however these lower doses did not result in increased REST mRNA expression (Fig. 2B), suggesting that some effects of EPO treatment may be dose-dependent. Ingenuity pathway analyses of the differentially expressed genes was highly enriched in pathways associated with neural development and neural protection (Fig. 2D and E), including “proliferation of neuronal cells” (p value 2.28 e-03), “regeneration of motor neurons,” (p value 5.34e-3) and “development of neurons,” (p value 5.49e-03) further supporting a role for EPO signaling in these essential processes.

### The differentially bound regions (DBR) have extensive overlap with active regulatory elements

**Figure 3:**
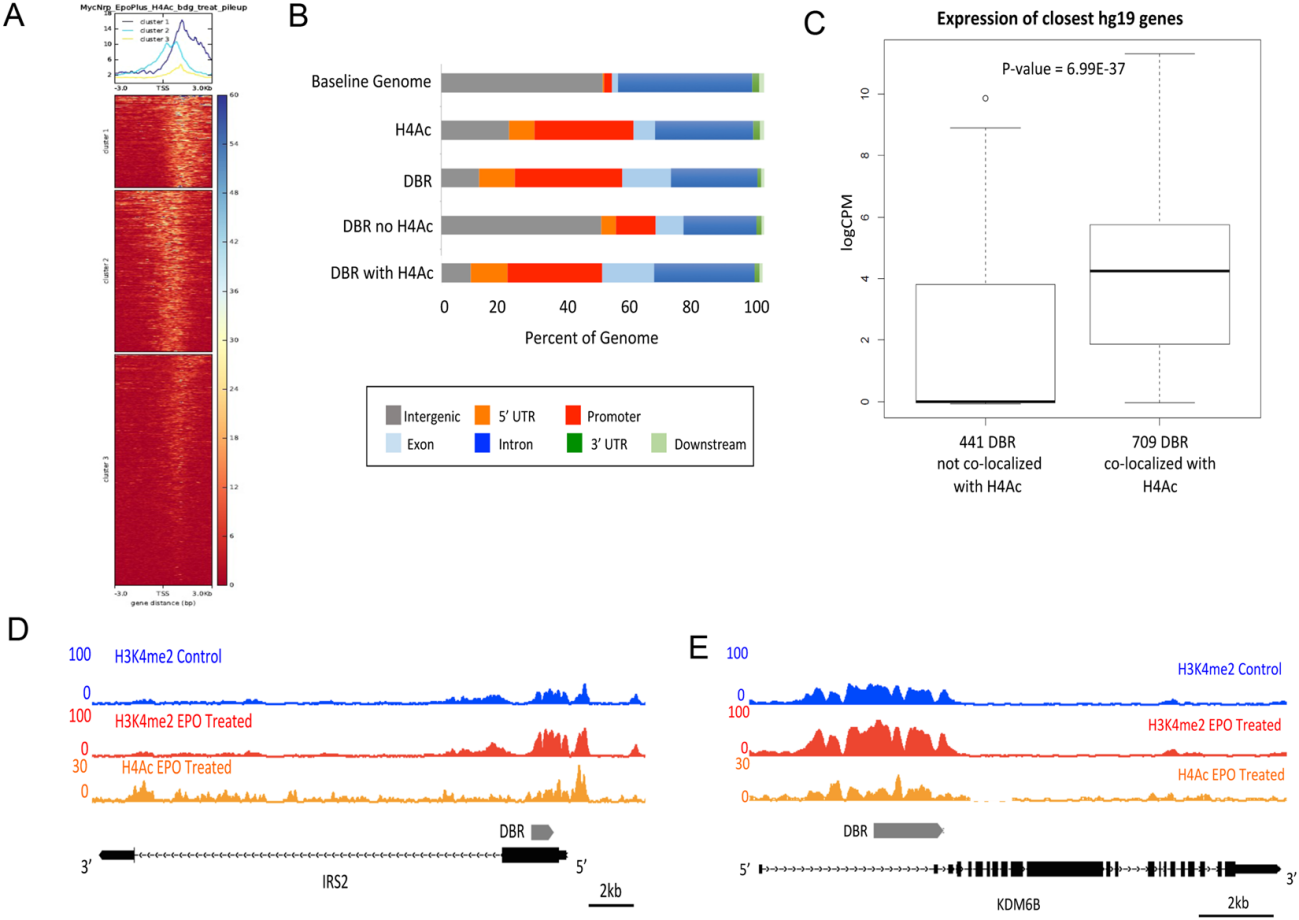
DBR co-localize extensively with regions of H4 Acetylation. (**A**) Heat map demonstrating the level of H4 Acetylation occupancy the 1150 DBR in EPO treated cells. (**B**) Relationship of DBR with and without co-localization of H4Ac to known genomic features. DBR that co-localize with H4Ac are located almost exclusively near protein coding genes, in stark contrast to DBR that do not localize with H4Ac, which are distributed more evenly throughout the genome. (**C**) Genes in close proximity (within 10 kb) of DBRs that co-localize with H4Ac are expressed at significantly higher levels than genes in close proximity of DBR that do not co-localize with H4Ac. (**D**) Example of H3K4me2 and H4Ac occupancy at the IRS2 locus. Grey bar represents DBR. (**E**) Example of H3K4me2 and H4Ac occupancy at the KDM6B locus. Grey bar represents DBR.

H3K4me2 marks both active and poised regulatory elements^38,44^. Acetylation of histone H4 (H4Ac) is more specifically associated with active regulatory elements^44^. To more clearly delineate if the DBR had significant overlap with regulatory elements that were active following EPO treatment, we performed ChIP-seq using a pan H4 acetyl antibody in EPO treated cells. The DBR had extensive overlap with regions of H4Ac occupancy, with ~62% of the DBRs co-localizing with a significant peak of H4Ac (Fig. 3A). The majority regions of H4Ac occupancy were located in close proximity to protein coding genes, and were enriched at TSS and promoter regions (Fig. 3B). DBR that did not co-localize with H4Ac were significantly more enriched in intergenic regions (Fig. 3B). Examples of DBR that co-localize with H4Ac are shown in Fig. 3D and E.

As regulatory elements marked by H4Ac often promote transcription, we analyzed gene expression near DBR with and without H4Ac co-occupancy. Genes located within 10 kb of DBR that had co-occupancy of histone H4Ac were expressed at significantly higher levels than genes located within 10 kb of DBR not associated with H4Ac (Fig. 3C, p = 6.9e-37). These results identify a group of regulatory elements that are active in EPO treated cells and raise the intriguing possibility that EPO signaling modulates usage of regulatory elements, such as enhancers, in neural progenitor cells.

### The differentially bound regions are associated with a complex network of transcriptional and epigenetic regulators

**Figure 4:**
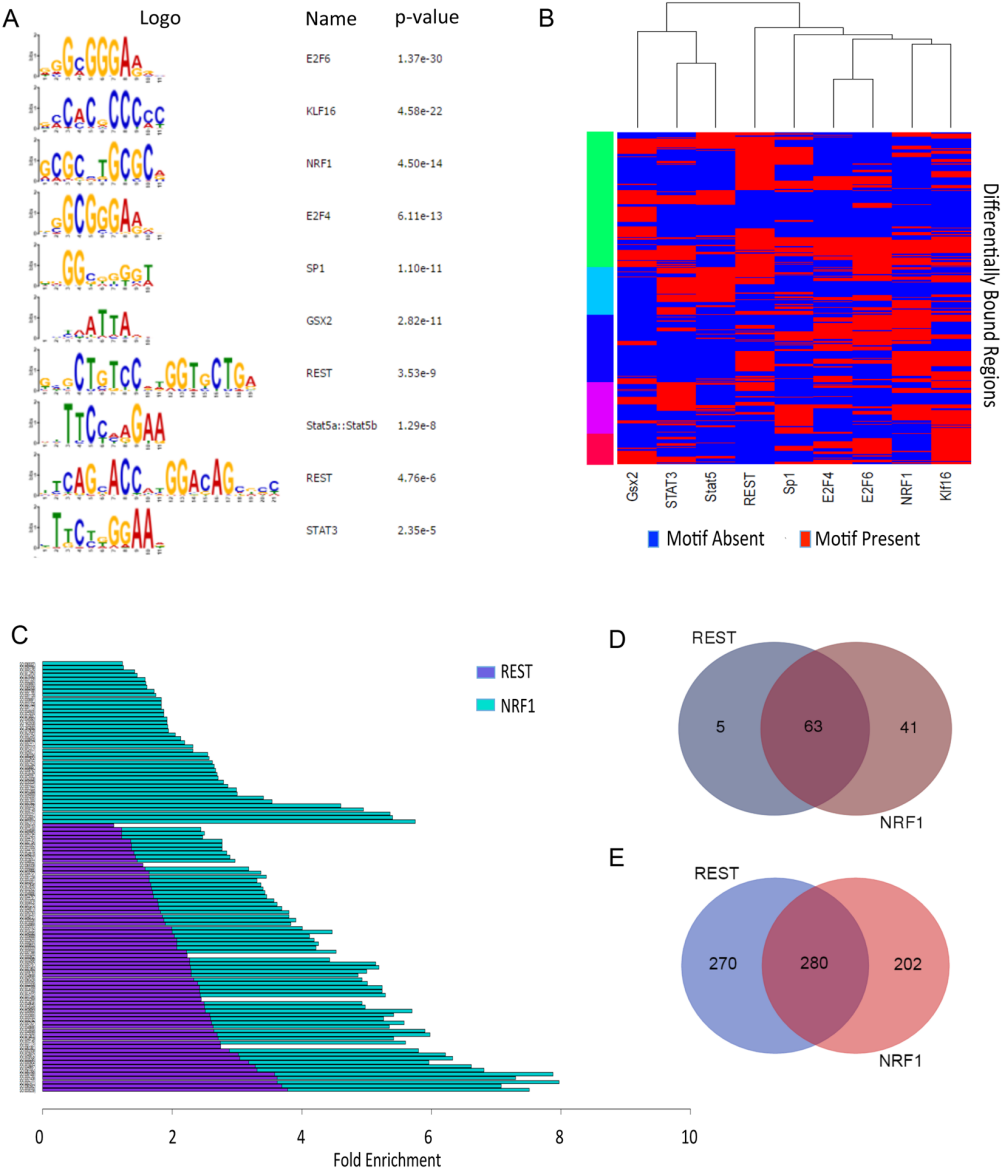
An extensive network of transcriptional regulators underlies the DBR. (**A**) Meme-ChIP analyses identified several overrepresented motifs in the DBR. (**B**) Heat map depicting occupancy of significantly enriched motifs at the 1150 DBR. Factors whose motifs were significantly enriched in the DBR are listed on the x-axis and the DBR are on the Y-axis. Blue indicates a motif for a listed factor is absent in the DBR and Red indicates that the motif for a listed factor is present in that DBR. Many DBR contain motifs for more than one factor. (**C**) Gene set enrichment analyses of genes within 10 kb of a DBR containing rest motif (purple) or NRF1 motif (green) identified significant enrichment for multiple pathways. The graph demonstrates considerable overlap between pathways identified in association with REST- and NRF1- containing DBR. (**D**) Venn diagram representing the number of DBR that contain motifs for REST and/or NRF1. Many DBR contain motifs for both factors. (**E**) Venn diagram demonstrating the number of genes within 10 kb of DBR containing motifs for REST and/or NRF1.

To gain insights into the signaling mediators and transcription factors promoting these epigenetic and transcriptional changes, we used meme-ChIP^45^ to interrogate the DNA sequences underlying the DBR for overrepresented DNA binding motifs. Several known mediators of EPO signaling were identified, including STAT5 and STAT3 (Fig. 4A,B, S4). Consistent with EPO’s role in promoting cellular proliferation^25,26^, motifs for E2F4 and E2F6, factors that are important for promoting cell cycle progression^46,47^, were also significantly enriched in the DBR (Fig. 4A,B). Motifs for a number of other transcriptional and epigenetic regulators were also overrepresented, including sp1, a general transcriptional activator^48^ and several factors important for neural development, specification, and maintenance, including GSX2, NRF1 and REST^43,49–54^. (Fig. 4A,B) Gata3 and NFKB have been previously associated with EPO signaling in neural cells^3,14,34,37^, however the DBRs did not have significant enrichment for the binding motifs of those factors.

We focused on DBR containing REST and NRF1 motifs because both factors are important for neural development and homeostasis, but to our knowledge have not been previously described to be downstream of EPO signaling in neural cells. NRF1 has a role in neural development and maintenance^54,55^, and it is an important regulator of cellular proliferation, mitochondrial biogenesis, proteasome function, and antioxidant and cytoprotective genes^56,57^. REST is an epigenetic modifier that also has a central role in neural development and has been associated with neuroprotection in a variety of settings^43,58^. We performed pathway analyses on the nearest genes assisted with DBR that contained either a STAT3, STAT5, REST or NRF1 motif. Although approximately half of the DBR were associated with a STAT3 or STAT5 motif, analyses of the genes associated with these DBR did not identify any specific pathways. In contrast, analyses of genes near DBR associated with REST motifs identified 68 pathways, many of which related to neural development or neurogenesis (Fig. 4C and S5). Similarly, pathway analyses of genes located near DBR associated with NRF1 motifs identified enrichment for 104 pathways, many of which related to neuronal survival, proliferation, or differentiation (Fig. 4C and S6). Some of the most highly enriched pathways were “spinal cord development” and “regulation of neuron differentiation” (Fig. 4C and S5).

There was a large sub-population of DBR that contained motifs for both REST and NRF1 (Fig. 4B and D). Interestingly, those DBR were frequently distinct from the DBR that contained motifs for STAT3/5, the canonical mediators of EPO signaling (Fig. 4B and S4). Consistent with the large subset of DBRs that contained motifs for both REST and NRF1, there was significant overlap in the identity of the genes located within 10 kb of a DBR containing either a REST or NRF1 motif (Fig. 4E) and significant overlap of the pathways associated with DBR containing these motifs (Fig. 4C, 5–6). The large number of DBR containing both REST and NRF1 motifs raise the interesting possibility REST and NRF1 may act in a cooperative manner downstream of EPO-signaling in the developing nervous system.

### The differentially bound regions have extensive co-localization with REST (NRSF) and NRF1

**Figure 5:**
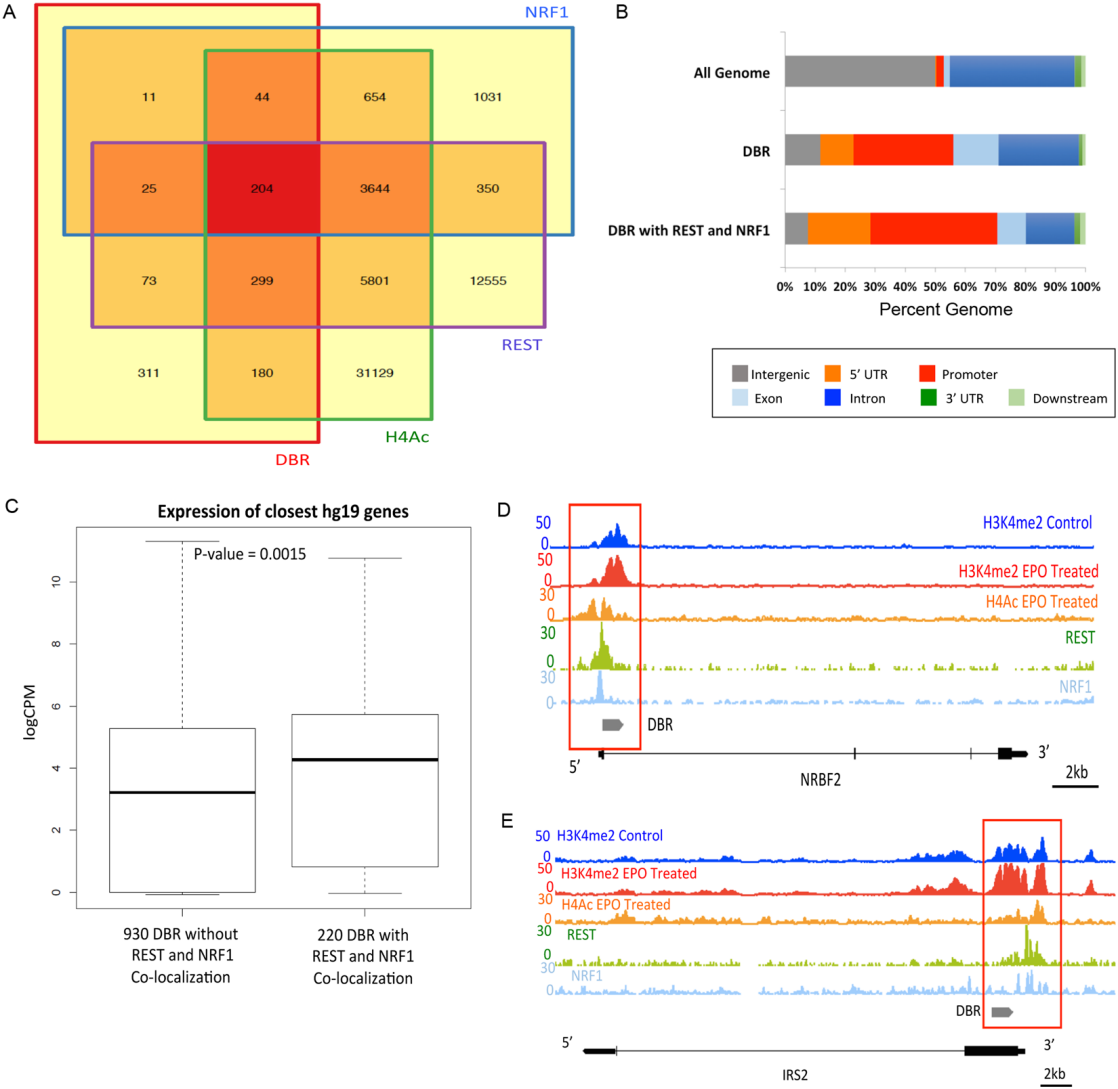
REST and NRF1 frequently co-localize with DBR. (**A**) Venn diagram depicting co-localization of DBR, H4Ac, NRF1, and REST. (**B**) Locations of DBR that co-localize with REST and NRF1 in relation to known genomic features. (**C**) Genes located near a DBR that contained an NRF1 and REST motif were expressed at significantly higher levels than genes located near DBR that lacked a motif for either factor. (**D**) Example of H3K4me2, H4Ac, REST and NRF1 occupancy at the NRBF2 locus. Grey bar represents the DBR. (**E**) Example of H3K4me2, H4Ac REST and NRF1 occupancy at the IRF2 locus. Grey bar represents the DBR.

We compared our DBRs to a published ChIP-seq data set of REST occupancy in human neural progenitor cells^59^. Consistent with our DNA motif analyses, 633/1150 (55%) of DBR overlapped with a site of REST occupancy (Fig. 5A). We also compared our DBR to a published ENCODE ChIP-seq track of NRF1 done in the EPO responsive neural cell line Sk-N-Sh^60^. There was extensive overlap of DBR with sites of NRF1 occupancy, with 277/1150 (24%) of DBR co-localizing with a peak of NRF1 occupancy (Fig. 5A).

Motif analyses demonstrated that NRF1 and REST motifs were frequently present in the same DBR (Fig. 4B and S4). Consistent with that data, there was frequent co-localization of REST and NRF1 ChIP-seq peaks at the DBR; 220/277 (79%) of DBR that co-localized with NRF1 also co-localized with REST. The vast majority of DBR that co-localized with both REST and NRF1 occupancy were located in promoters/5′UTRs (~62%), and introns (~16%), with relatively few sites located in intergenic regions (~8%; Fig. 5B). Genes located near DBR that overlapped sites of REST and NRF1 occupancy had significantly higher expression than genes in close proximity to DBR that did not co-localize with sites of NRF1 or REST. (Fig. 5C, p value = 0.0015) Examples of DBR that co-localize with REST and NRF1 are shown in Fig. 5D–E. Pathway analyses of the genes near DBR that co-localized with NRF1 and REST identified enrichment for “pons maturation” and “N-glycan processing,” which is an important regulator of normal neural development and plays an important role modulating neuronal excitability^61^.

### EpoR deletion is associated with altered expression of key transcriptional and epigenetic regulators in the developing nervous system

**Figure 6:**
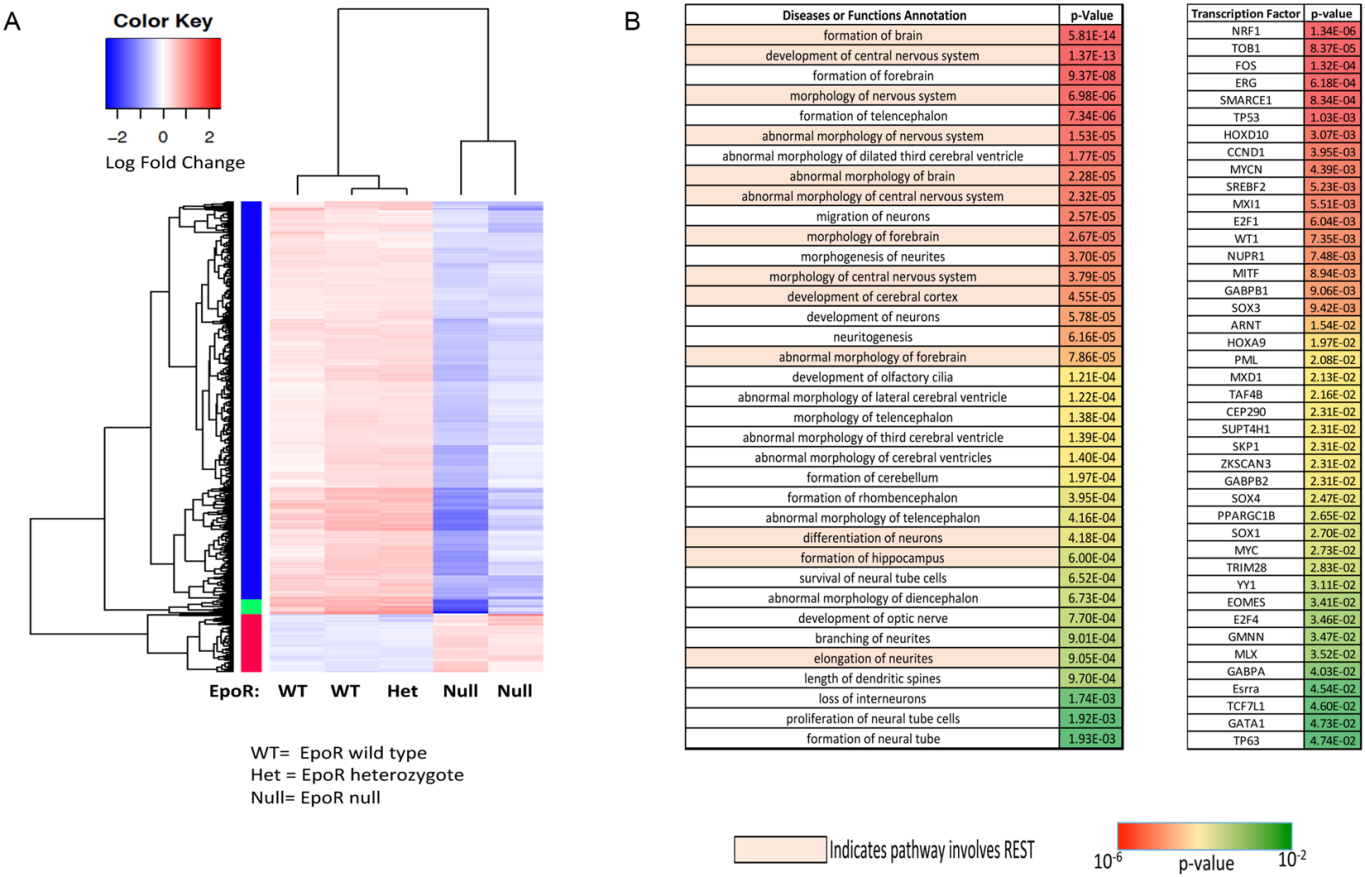
Transcriptome analyses of EpoR-null and control cells. (**A**) Heat map of genes differentially expressed in neural tubes isolated from EpoR null and littermate control E9.0 embryos. (**B**) Left panel: Diseases or Functional Annotations identified through Ingenuity Pathway Analyses of differentially expressed genes. Pathways highlighted in orange include REST. Right panel: Transcription factors predicated to be upstream of genes differentially expressed between EpoR-null and littermate control neural tubes. NRF1 is the most significantly enriched transcription factor predicted to be upstream of the differentially expressed genes.

As immortalization and cell culture can introduce significant artifact, we sought to analyze the function of EPO signaling in primary, uncultured neural progenitor cells. We therefore performed RNA-seq on the neural tubes isolated from E9.0 (Embryonic Day 9.0; E9.0) EpoR-null and littermate control embryos. The E9.0 time point was chosen because EpoR is highly expressed in the developing nervous system of control embryos at E9.0^6^, the embryos have not yet developed significant anemia from lack of EpoR signaling^62^, and the neural tube at E9.0 is a rich source of neural progenitor cells^63,64^. In total, 574 genes were differentially expressed in the EpoR-null neural tubes compared to neural tubes from littermate controls (p < 0.001, FDR < 0.05; EpoR+/+ n = 2, EpoR+/− n = 1, EpoR−/− n = 2; Fig. 6).

Compared to controls, EpoR-null cells had lower expression of genes that promote cell cycle progression, such as ccnd2 (log fold change −0.69, p < 10^−3^, FDR < 10^−2^) and Trim71 (log fold change −1.8, p < 10^−14^, FDR < 10^−10^). The EpoR-null cells also had lower expression of multiple genes associated with DNA damage response, including ATM (log fold change −0.85, p < 10^−5^, FDR < 10^−5^), ATR (log fold change −0.61, p < 10^−2^, FDR < 0.05), and cdk12 (log fold change −0.65, p < 10^−2^, FDR < 10^−2^) and altered expression of genes regulating apoptosis including mdm4 (log fold change −1.4, p < 10^−7^, FDR < 10^−7^), hipk2 (log fold change −2.0, p < 10^−12^, FDR < 10^−12^), and myst3 (log fold change 1.1, p < 10^−7^, FDR < 10^−4^). These data further support a role for EPO signaling in promoting the survival and proliferation of fetal neural progenitor cells.

Consistent with our *ex vivo* data in human fetal neural progenitor cells, the EpoR-null cells had significantly lower expression of REST (log fold change −1.5, p < 10^−7^, FDR < 10^−4^) than wild type littermate controls. Ingenuity pathway analyses of the differentially expressed genes identified multiple functional annotations involving nervous system development including “formation of brain” (p = 5.8e-14) and “development of central nervous system” (p = 1.37e-13) as well as “survival of neural tube cells” (p = 6.25e-4). Many of these pathways included REST (Fig. 6B). Also consistent with our *in vitro* data, IPA analyses identified NRF1 as the most enriched transcription factor upstream of the differentially expressed genes (Fig. 6B, p = 1.34e-6). Nrf1 regulates proteasome gene expression and the expression of genes involved in mitochondrial biogenesis^55,56^. The EpoR-null cells had modest but significant decreases in the expression of multiple genes important for mitochondrial biogenesis (Table 2S) as well as the proteasomal subunit PSMB4 (log fold change −0.5, p < 0.005, FDR < 0.05). These data further support a link between EPO signaling and the REST- and NRF1-mediated transcriptional programs.

## Discussion

Erythropoietin signaling is important both for normal neural development and recovery from neural injury^4,14,15,25^. Our data demonstrates that EPO treatment is associated with changes in the epigenetic landscape of human fetal neural progenitor cells. These changes were highly enriched at gene promoters and putative regulatory elements (Figs 1D, 3B). Analyses of the DNA sequences underlying these regions revealed overrepresentation of motifs known to be associated with EPO signaling, including STAT3 and STAT5, as well as motifs of factors not previously described as being downstream of EPO signaling in neural cells, including REST and NRF1 (Fig. 4A,B, S4). NRF1 and REST were subsequently found to extensively co-localize with the DBRs (Fig. 5A). Analyses of the genes closest to the DBR identified many pathways relating to neural development and neural protection. Global transcriptome analyses of primary EpoR-null neural tubes from E9.0 embryos revealed changes in the expression of genes that regulate the proliferation, maturation, and survival of neural progenitors and further suggested a connection between EPO signaling, REST, and NRF1, although the specific mechanism of how these factors work together remains to be uncovered.

NRF1 is a cap n collar transcription factor that regulates the expression of anti-oxidant, cytoprotective, mitochondrial, and proteasome genes^55,57,65,66^. NRF1 activates expression of target genes by heterodimerizing with small Maf proteins and binding to either antioxidant response element (ARE) or Maf Recognition Elements (MAREs)^56,67,68^. NRF1 is highly expressed during erythropoiesis^69^ and in the developing nervous system^70^. Our data suggests that NRF1 is a mediator of EPO signaling in neural progenitor cells. Intriguingly, there are many parallels between the phenotype of NRF1 knockout and EpoR knockout mice, both of which are embryonic lethal mid-gestation due to severe anemia^2,71^. Targeted deletion studies have demonstrated that NRF1 is essential for cellular homeostasis in a number of biologic contexts, including the brain^54,55,72,73^. The role of NRF1 in the setting of recovery from hypoxic-ischemic insult is less clearly delineated, however cell culture models attempting to recapitulate hypoxic-ischemic conditions suggest that increased NRF1 expression is associated with neural protection^66^ and increased NRF1 expression was associated with neuronal survival in mice after excitotoxic brain injury^74^.

REST is an epigenetic modifier that has an important role in the regulation of neural cell maturation, function, and survival^43,58,75–78^. REST is highly expressed in the developing nervous system, peaking in the second trimester^8^. REST expression decreases near the time of birth but eventually expression increases again in the sixth to seventh decade of life^58^. Elevated levels of REST in the human brain are associated with preservation of cognitive function during aging^58^. In addition, increased expression of REST is neuroprotective in animal models of Parkinson’s disease, epilepsy, and fetal alcohol syndrome^79–81^. REST binds to the RE1 sequence and via zinc fingers exerts its effects on gene expression by recruiting cofactors such as CoREST^82^, the histone deacetylase mSin3a^83^, the histone H3 lysine 9 methylase G9a, and members of the SWI/SNF family of chromatin remodelers^76,84^. The complex interactions between REST and its co-repressors mediates short-and long-term gene repression in a both cell type- and developmental stage- specific manner^43^.

Our data connect EPO signaling to the level of REST expression both *in vitro* and *in vivo* (Figs 2A–C and 6) and implicate REST as a downstream mediator of EPO signaling (Figs 4A,B and 6). Of note, REST can also occupy repressed regions devoid of active epigenetic marks, such as H3K4me2 and H4Ac^84–88^. Increased REST occupancy at those sites may be also be important mediators of EPO’s effect in the developing nervous system, but those sites cannot be evaluated with our current data sets. Somewhat unexpectedly, EPO treatment was associated with increased H3K4me2 occupancy and higher levels of gene expression near REST-associated DBR, despite the fact that REST is generally considered to be a transcriptional repressor. There are several possible explanations for this observation. This first is that REST can occupy regions that are “poised” for activation, repressing gene expression until a stimulus causes it to be evicted^43,84,88^. The second is that REST is well described to co-localize with active histone modifications and its function at those sites is determined by co-factor occupancy^84,89^.

Our data demonstrate extensive co-localization of NRF1 and REST at DBR, suggesting that there may be a functional relationship between these factors. Published data regarding functional interactions between NRF1 and REST are limited, however a recent study done in neural progenitor cells demonstrated that REST can facilitate NRF1 occupancy by promoting local DNA hypomethylation^90^. Together with our data, this raises the possibility that the increase in REST expression following EPO treatment (Fig. 2A–C) facilitates NRF1 binding and activity at a subset of DBRs. The decreased expression of NRF1 target genes following EpoR deletion in primary murine neural cells (Table S2) is consistent with this model. EPO likely exerts its neuroprotective effects via a number of different mechanisms. We speculate that in neural progenitor cells, one of those mechanisms is increased REST expression, which then promotes the activity of NRF1, and the expression of cytoprotective and antioxidant genes that are likely to be beneficial in the setting of hypoxic ischemic insult. Future studies further elucidating the relationship between the epigenetic landscape, REST, NRF1, and gene expression are likely to provide valuable insights into the molecular mechanisms regulating neurodevelopment and recovery from neural injury.

The signaling pathways, epigenetic, and transcriptional changes downstream of EpoR are incredibly complex. We performed our *in vitro* studies following 24 hours of EPO treatment to assess steady state changes in the chromatin landscape and transcriptome, similar to those that might occur following exogenous treatment with EPO for neuroprotection. The signaling pathways downstream of EPO receptor signaling are highly dynamic, with phosphorylation of signaling mediators such as STAT5 peaking shortly after EPO receptor activation^28^. It is likely that serial assessment of epigenetic and transcriptional changes beginning shortly after EPO administration would reveal additional transcriptional and epigenetic modifications downstream of EpoR activation. Similarly, there are likely additional transcriptional and epigenetic changes that occur following EPO administration in the context of stress environments such as hypoxia, nutrient deprivation, glutamate toxicity, or hypoxia-ischemia reperfusion injury. Attempting to recapitulate these insults *in vitro* can be somewhat artificial and can significantly confound analyses of the effect of EpoR signaling. For these reasons, we performed our *ex vivo* genomic studies in neural progenitors with the addition of EPO as the only variable. In addition, the signaling pathways, epigenetic, and transcriptional changes downstream of EpoR are also likely to be cell type- and developmental stage- specific. Future studies examining the effects of EPO signaling in other cell types that promote recovery from neurologic injury, or studies done in the setting of stress conditions, will likely provide important insights into the molecular mechanisms underlying the neuroprotective effects of EPO treatment.

In conclusion, our data provide an unbiased assessment of the effect of EPO signaling on the epigenome and transcriptome of fetal human neural progenitor cells, identifying REST and NRF1 as associated with EPO signaling. These results were further supported by global transcriptome analyses of primary EpoR-null neural tubes. Together, these data provide novel insights into the molecular mechanisms downstream of EPO signaling in the developing brain.

## Methods

### Ethics Statement

IRB approval was obtained for studies using de-identified human tissues, University of Rochester Subjects Review Board (RSRB) #00024759. The University of Rochester’s Committee on Animal resources approved all experiments utilizing mice (UCAR 101396). All experiments were performed in accordance with relevant guidelines and regulations.

### Isolation, Immortalization, and Culture of Human Fetal Neural Cells

Human neural progenitors were isolated from the ventral mesencephalon of 10-week-old fetal brain, and transduced with v-myc-expressing lentivirus as previously described^91,92^. These immortalized hVM1 neural progenitors demonstrated extensive self-renewal in the presence of 20ng/ml bFGF and EGF, and can be differentiated into TuJ1, LMX1A and tyrosine hydroxylase (TH) expressing neurons by pretreatment with 2 µM purmorphamine and 20ng/ml sonic hedgehog (Shh C24Il) followed by withdrawal of FGF2 and EGF, and addition of 10 nM all-trans retinoic acid (ATRA), 10ng/ml BDNF and 10ng/ml GDNF in Neurobasal B-27 medium. Mitogen withdrawal and treatment with 10% fetal bovine serum or 20ng/ml bone morphogenetic protein-4 induced differentiation into GFAP expressing astroglia^93^. Following establishment of the cultures, Myc-immortalized neural progenitor cells were grown in DMEM/F12 with N2 supplement (Fisher), 20ng/ml EGF and FGF (Miltenyi Biotec, CA), with gentamycin in lamin (1.25ng/cm^2^) coated flasks. The cells were passaged every 4 days. Cells were treated with EPO (Amgen; 10,000 U/ml) 10ug/ul for 24hrs prior to ChIP-seq and RNA-seq experiments. For the quantitative PCR validation of REST mRNA expression, cells were treated with various concentrations of EPO (0, 0.4, 2, or 10 U/ml) for 24 hours prior to RNA isolation. For the validation of REST protein expression, cells were treated with EPO 10U/ml for 24 hours prior to protein isolation.

### Animals, Generation of Timed Embryos, and RNA Preparation

The University of Rochester Committee on Animal Resources approved all experiments involving animals. EpoR+/− mice^3^ were bred to generate timed pregnancies. Mice were bred overnight and vaginal plugs checked after 12 hours (Embryonic Day 0.5; E0.5). At E9.0, the pregnant dam was anesthetized and sacrificed via cervical dislocation and the embryos dissected for further analyses. Embryos were genotyped as previously published^4^. Neural tubes were obtained using a combination of manual and enzymatic dissection. RNA was isolated using the PicoPure RNA Isolation kit (ThermoFisher).

### Antibodies for Western Blot and Chromatin Immunoprecipitation Assays

Antibodies used in this study included H3K4me2 (Abcam), H4Ac (Abcam), and REST (Abcam).

### ChIP Sequencing and Annotation

Chromatin Immunoprecipitation and library preparation were done as previously described^94,95^. Library quality was evaluated on a Bioanalyzer and sequencing was performed on a HiSeq. 2500 Rapid Run to obtain 1 × 50 bp reads. Each library was sequenced to at least 25 million raw reads. Quality control was executed on sequenced reads using Trimmomatic (v0.32). All quality reads were aligned to hg19 using Bowtie (-m 1) to exclude multi-mapping reads. Aligned reads were de-duplicated using picard-tools before peaks were called for each replicate using MACS2. Significant peaks were annotated with their genomic location using CEAS (v1.0.2). The bedtools (v2.25.0) intersect function was used to identify H3K4me2 marks shared between replicates for each treatment.

### Identification of Differentially bound regions

Alignment files were converted to the bed format using bedtools bamtobed function for each replicate in both treatments. Differentially bound regions between treatments were identified using DiffReps-nb^41^. A conservative significance threshold was defined as an adjusted p-value < 0.001 to identify 1,150 significant differentially bound regions between EPO treated and control samples. Differentially bound regions were annotated with their genomic locations using CEAS^96^. Heat maps showing the differences in binding affinity between the 1,150 regions were created using the compute-matrix and plotHeatmap functions within deepTools (v2.2.4).

### Quantitative ChIP Validation of DBR

ChIP assays were performed as previously described^97,98^, with approximately 10 million cells used for each assay. Briefly, DNA was cross-linked to DNA binding proteins using 1% formaldehyde. The cells were lysed and the DNA isolated and sonicated into ~200 bp fragments using a Diagenode Bioruptor. The DNA-protein complexes were immunoprecipitated with an antibody to H3K4me2 (Abcam). DNA-protein complexes were recovered with protein G magnetic beads (Invitrogen). Immunoprecipitated DNA was subjected to quantitative PCR and enrichment compared to total input control calculated as previously described^97,98^. Primers are available on request.

### Co-localization with publically available datasets

Publically available data sets were downloaded from Gene Expression Omnibus (GEO) for REST and NRF1 from the following accession numbers: GSM1010804 and GSM1003630. Co-occupancy, in terms of overlapping marks and peak intensities were evaluated between the 1,150 DBR and the transcription factors of interest. The intersect function of bedtools was used to identify co-located regions between each mark and the differentially bound regions. The heat map visualizations displaying co-location and intensities within the shared regions were constructed using deepTools compute-matrix and plotHeatmap functions.

### RNA-sequencing and Differential Expression Analyses

RNA quality was assessed with an Agilent Bioanalyzer (Sana Clara, CA, USA) prior to polyA selection and library preparation according to Illumina instructions. Libraries were subjected to 65 bp single end sequencing on the Illumina HiSeq. 2500. Library quality was evaluated on a BioAnalyzer and sequencing was performed on a HiSeq. 2500 Rapid Run to obtain 1 × 100 bp reads. Each library was sequenced to at least 20 million raw reads. Quality control was executed on sequenced reads using Trimmomatic (v0.32). All quality reads were aligned to hg19 using Tophat (v2.0.1)^99^ and read counts were quantified using HTSeq (v0.6.1). Replicate quality and relatedness were evaluated in R using spearman correlation, multidimensional scaling, and principle component analysis. All replicates were library size normalized using the trimmed mean of M-values (TMM) and differential expression was executed following standard procedures using the exactTest function within the edgeR manual. Heat maps were generated using the heatmap.2 function in the gplots package. All genes with a fold change greater than 1.5 between Epo treatments were submitted for pathway analysis using Ingenuity Pathway Analysis (v01–07). The 1,150 DBR were extended 10 kb and associated with their nearest genes using the closest function in bedtools. Expression values for the nearest genes were assigned based on the RNA-seq data and box and whisker graphs were generated in R for the Epo treatments.

### Motif enrichment analysis

Motif enrichment analysis (using MEME-ChIP^45^) was conducted on the 1,150 DBR after using RepeatMasker (v4.0.6) to mask any repetitive regions.

### Availability of data and materials

All sequencing data reported in this paper have been deposited in GEO, accession number GSE99372.

## Electronic supplementary material

Supplementary Data
