# Supplementary material for: Erythropoietin Signaling Regulates Key Epigenetic and Transcription Networks in Fetal Neural Progenitor Cells
Source: Sci Rep. 2017 Oct 30;7:14381. doi: 10.1038/s41598-017-14366-0 (PMC5662632; doi:10.1038/s41598-017-14366-0)
Supplement: Supplementary file 1 — Supplementary Data [file 41598_2017_14366_MOESM1_ESM.pdf]

Supplemental Data for:

Erythropoietin Signaling Regulates Key Epigenetic and Transcription Networks in Fetal Neural Progenitor Cells

Christina Sollinger, * Jacquelyn Lillis, + Jeff Malik, * Michael Getman, * Chris Proschel, ¶ and Laurie Steiner*§

*Department of Pediatrics, University of Rochester, Rochester, New York, USA
+Functional Genomic Center, University of Rochester, Rochester, New York USA
¶ Department of Biomedical Genetics, University of Rochester, Rochester, New York, USA

Supplemental figure legends

Figure S1. Spearman correlation heat maps showing global sample relatedness for the H3K4me2 replicates.

Figure S2. Validation of H3K4me2 differentially bound regions (DBR) using quantitative ChIP.

Figure S3. Spearman correlation heat maps showing global sample relatedness for the RNA-seq replicates.

Figure S4. Heat map demonstrating of occupancy of STAT3, STAT5, NRF1, and REST in the 1150 DBR.

Figure S5. Pathway analysis of genes associated with DBR containing a REST motif.

Figure S6. Pathway analysis of genes associated with DBR containing an NRF1 Motif.

Figure S7. Full image of REST western blot shown in Figure 2C.

Table S1. Quality metrics of ChIP-seq studies.

Table S2. EpoR deletion is associated with changes in the expression of mitochondrial genes.

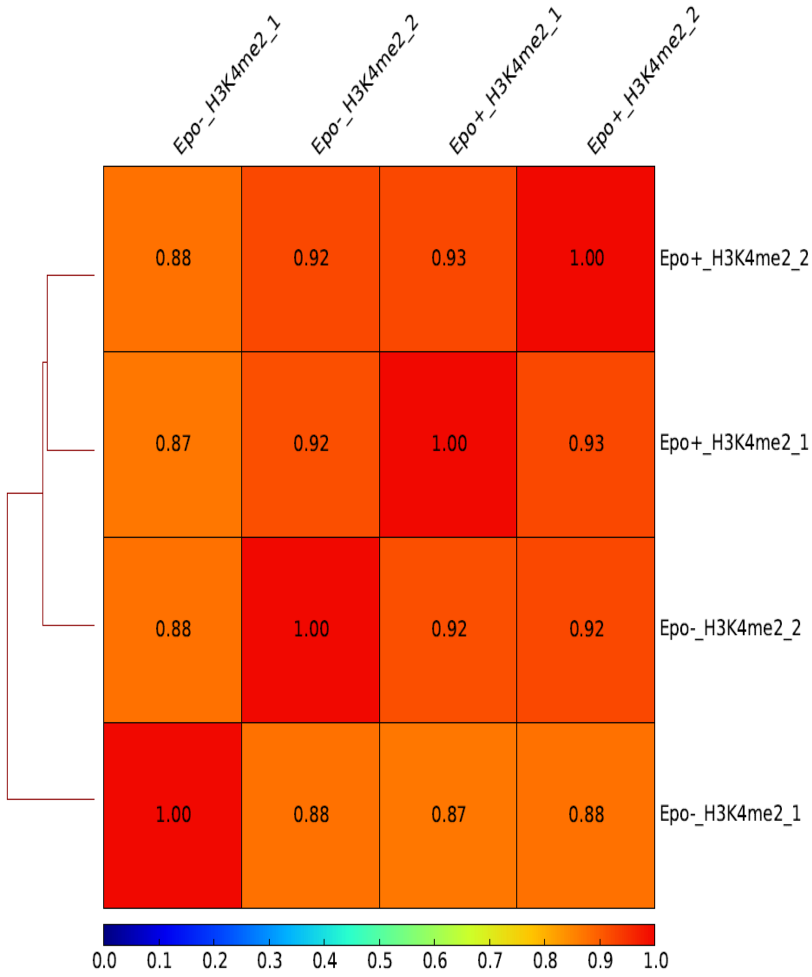

Figure S1. Spearman correlation heat maps showing global sample relatedness for the H3K4me2 replicates.

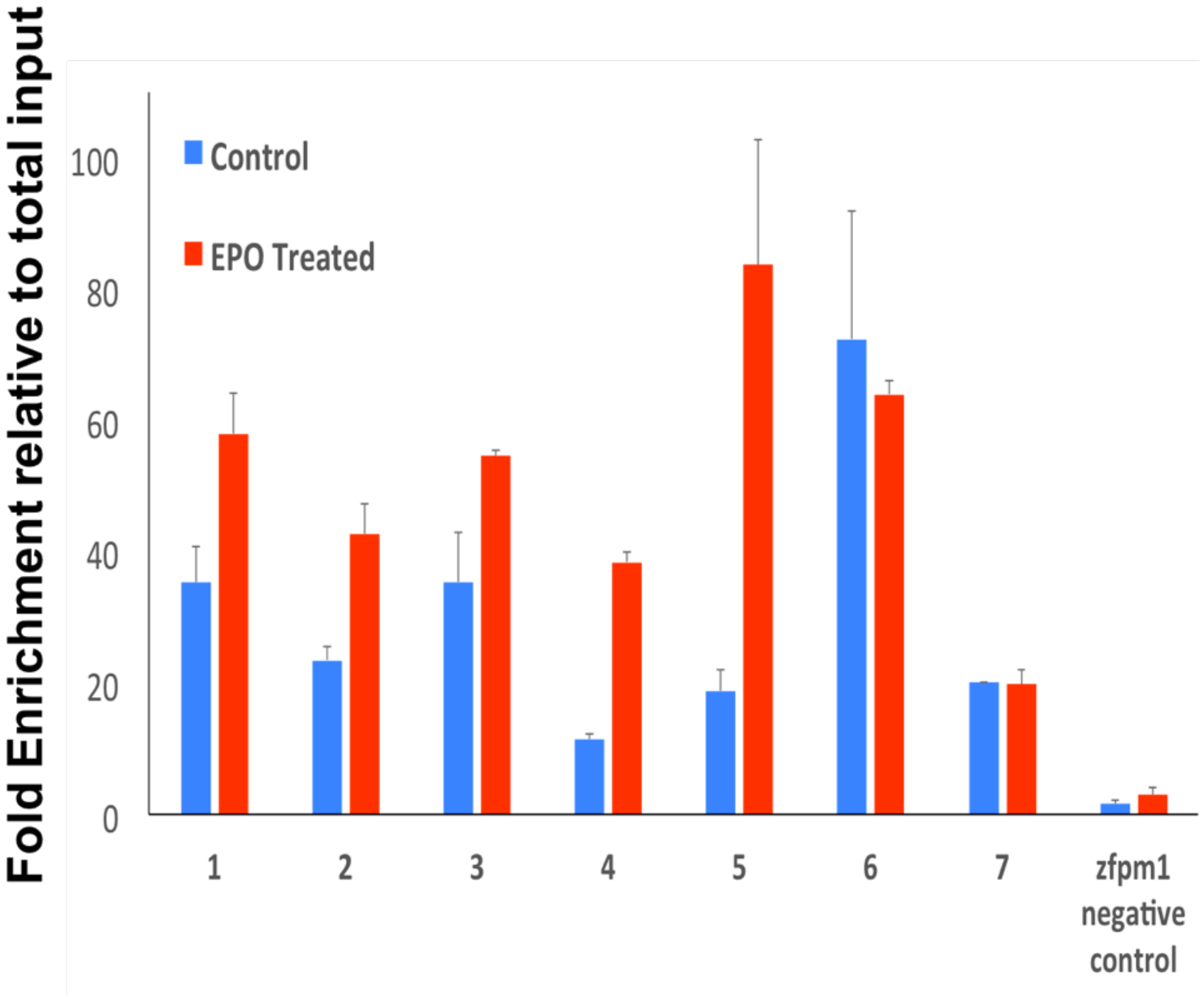

Figure S2: ChIP-qPCR validation of differentially bound regions.

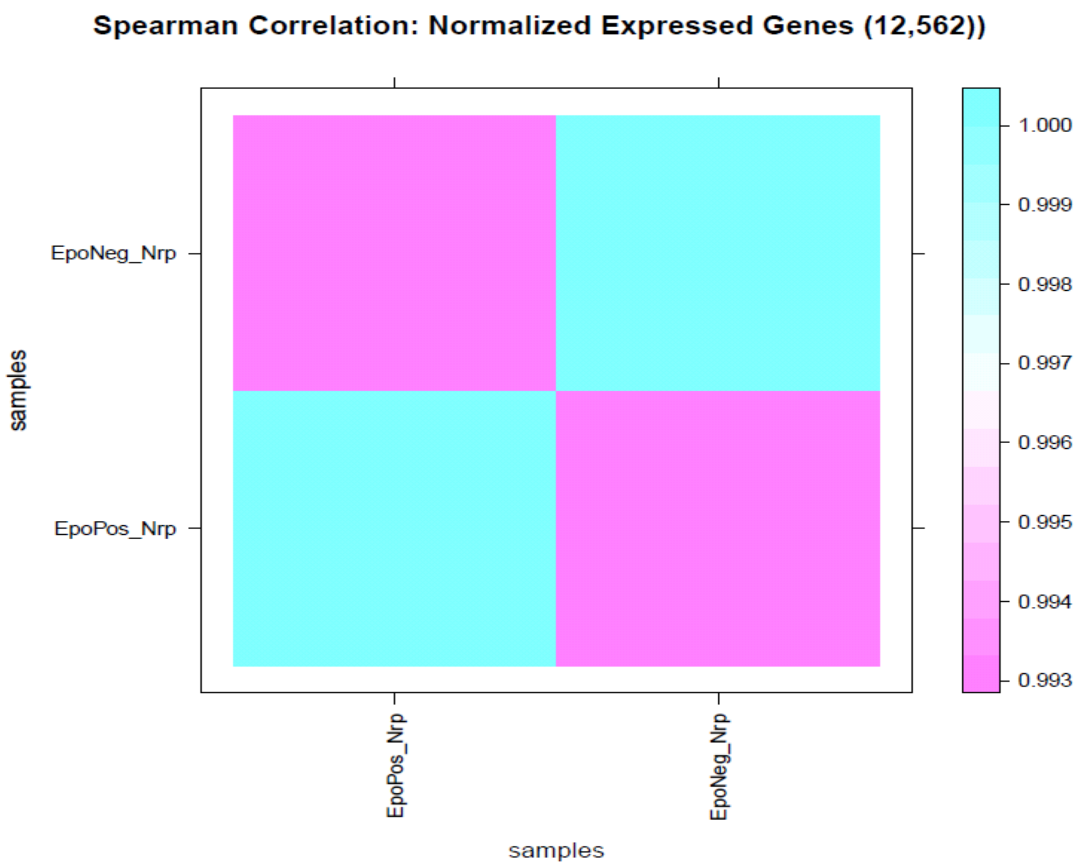

Figure S3. Spearman correlation heat maps showing global sample relatedness for the RNA-seq replicates.

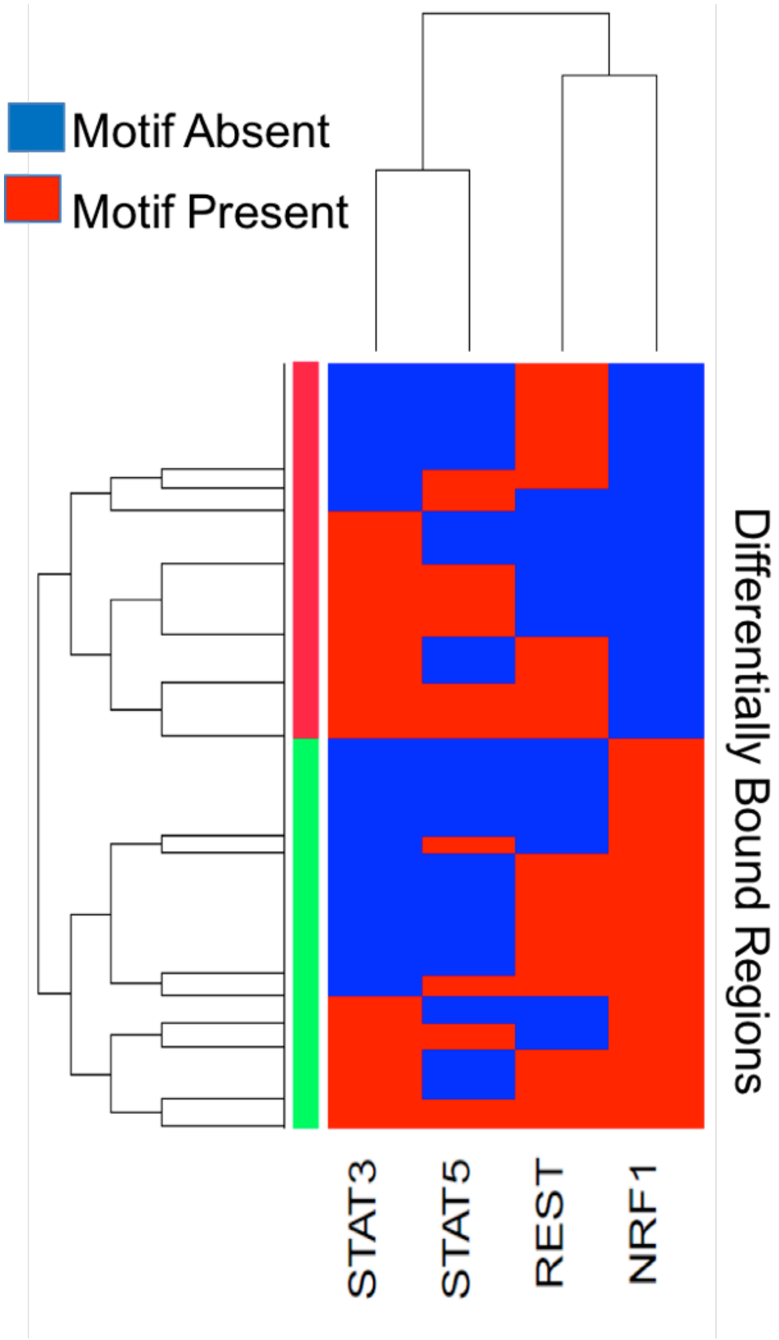

Figure S4. Heat map of DBR that contain STAT3, STAT5, REST, or NRF1 motifs.

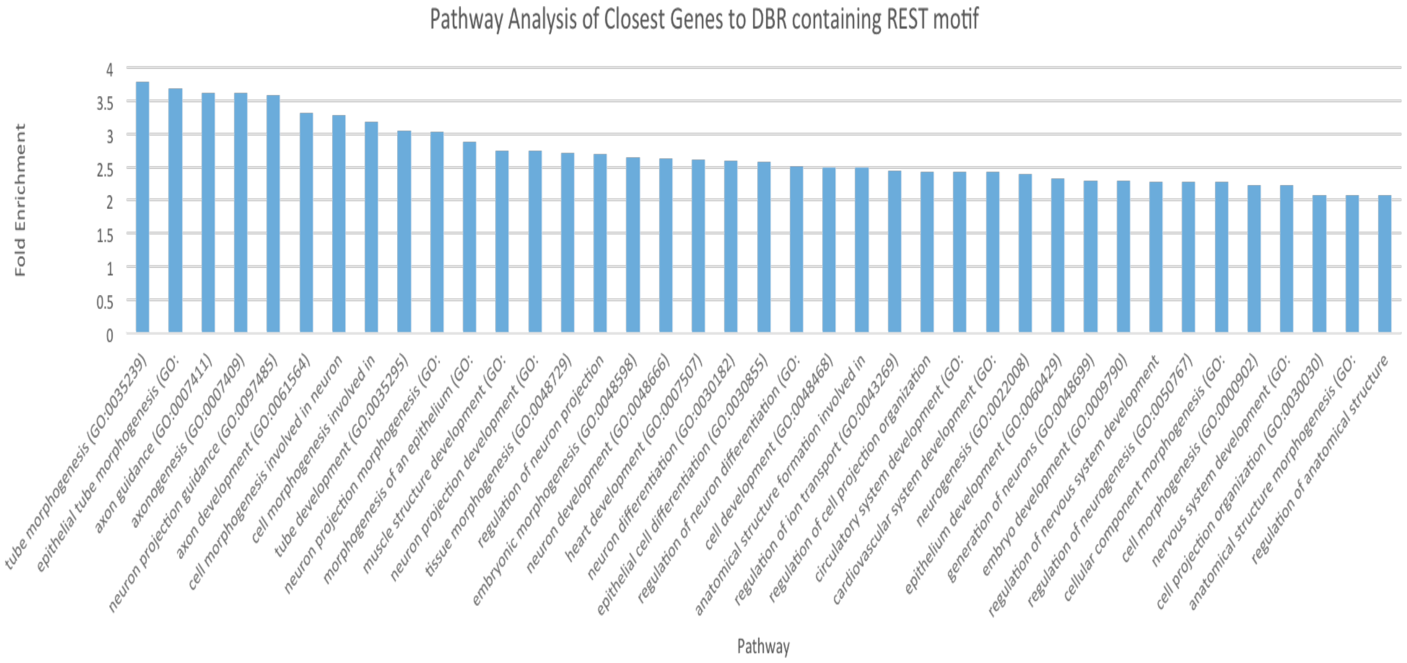

Figure S5: Pathway analyses of closest genes to DBR containing REST motif.

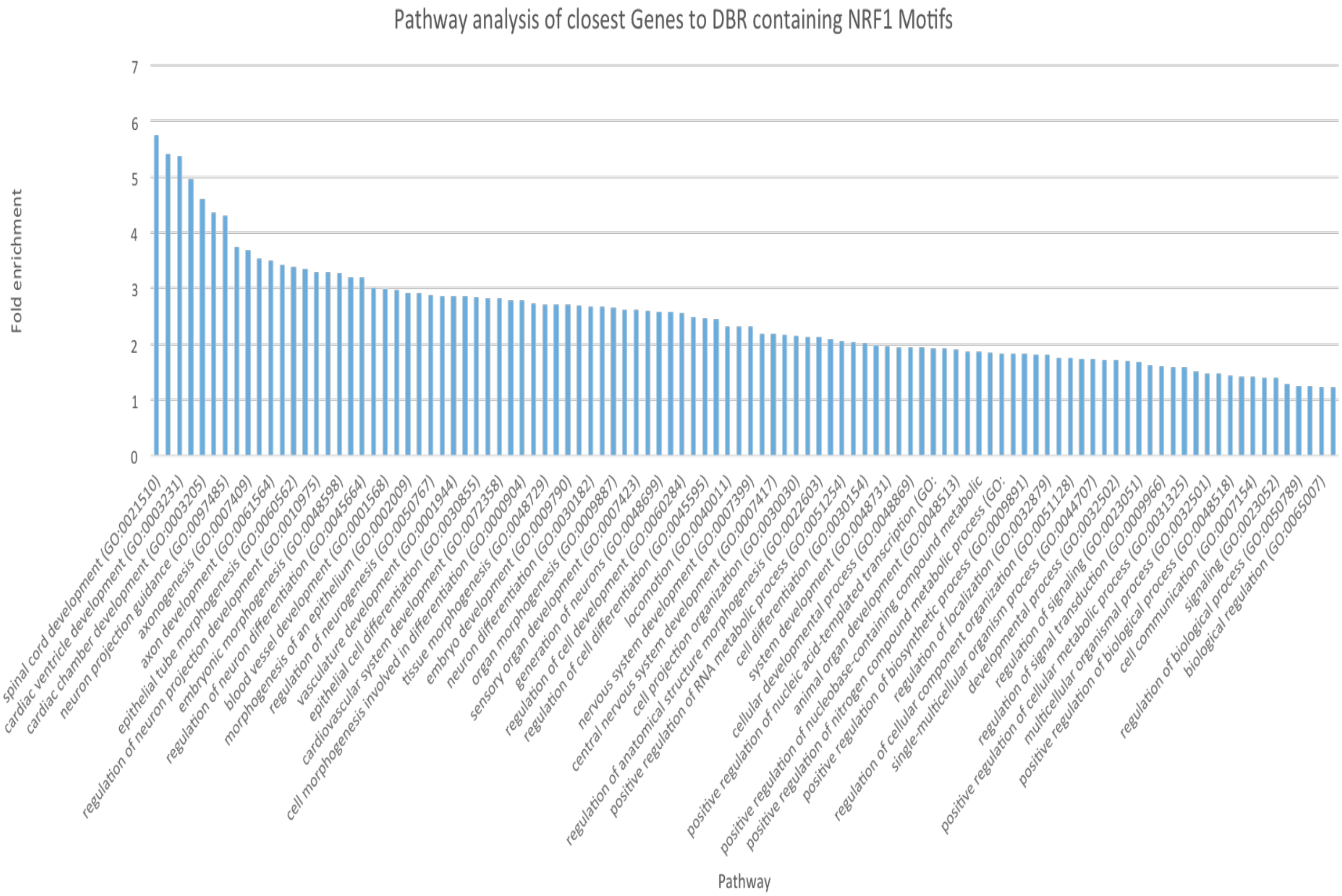

Figure S6: Pathway analyses of closest genes to DBR containing an NRF1 motif.

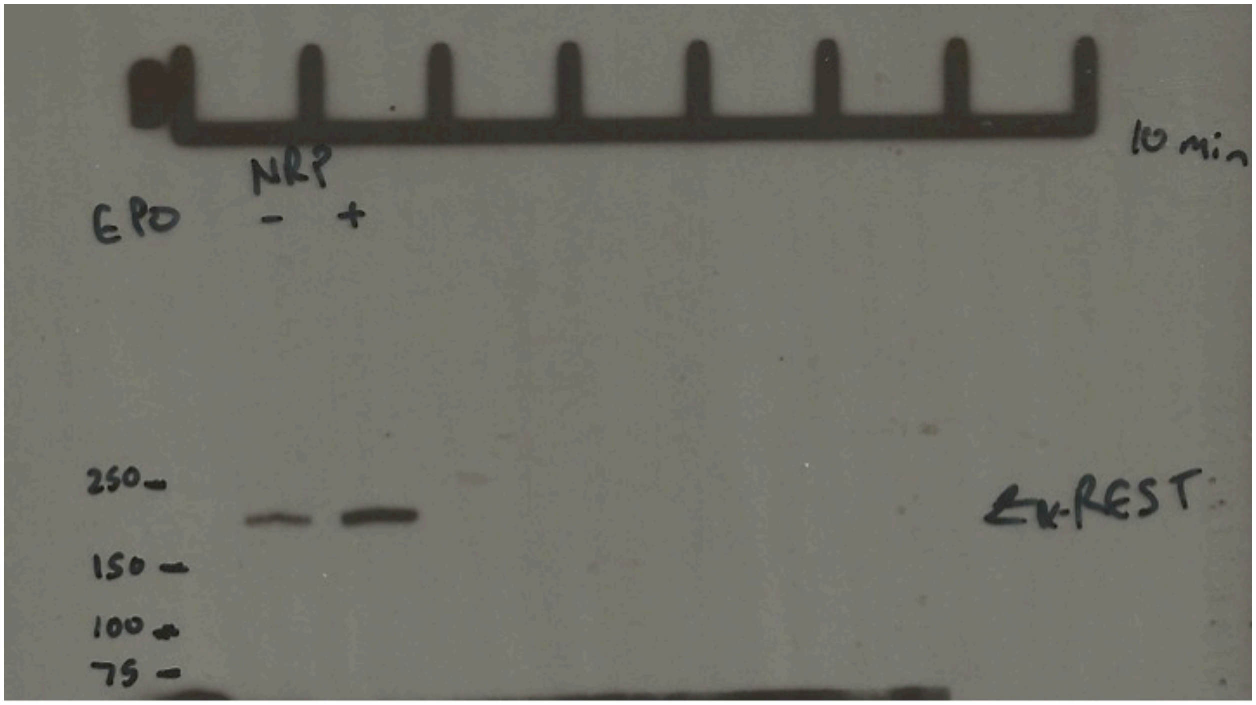

Figure S7. Full image of REST western blot shown in Figure 2C.

	bowtie						picard-tools MarkDuplicates		
Name	raw reads	mapped	%aligned	failed	multimappers	%multimapper	UNPAIRED_READS_EXAMINED	UNPAIRED_READ_DUPLICATES	PERCENT_DUPLICATION
MycNrp_EpoMinus_H3K4me2_1	25061140	20171364	80%	800140	4089636	16%	20171364	990936	5%
MycNrp_EpoMinus_H3K4me2_2	34008734	27282655	80%	1069413	5656666	17%	27282655	726231	3%
MycNrp_EpoPlus_H3K4me2_1	28661485	23453603	82%	784369	4423513	15%	23453603	1025175	4%
MycNrp_EpoPlus_H4Ac_1	26106491	20796414	80%	766607	4543470	17%	20796414	695904	3%
MycNrp_EpoPlus_H3K4me2_2	34868886	28586947	82%	952441	5329498	15%	28586947	983125	3%
MycNrp_EpoPlus_H4Ac_2	24401134	19431046	80%	724691	4245397	17%	19431046	488568	3%
MycNrp_EpoMinus_Control_1	24915346	19098401	77%	972083	4844862	19%	19098401	472582	2%
MycNrp_EpoPlus_Control_1	32740957	25390179	78%	1200720	6150058	19%	25390179	281840	1%

Table S1: ChIP-seq Quality Metrics

	Log Fold Change	PValue	FDR
Cox5a	-0.5	0.0017	0.04
Cox5b	-0.6	0.0005	0.02
Cox6a1	-0.5	0.0024	0.05
Cox6c	-0.6	0.0001	0.01
Cox7a2	-0.5	0.0010	0.03
Ndufb5	-0.6	0.0011	0.03
Uqcrh	-0.5	0.0006	0.02
Ndufb8	-0.6	0.0009	0.03
Ndufa3	-0.7	0.0013	0.03
Atp5g3	-0.5	0.0014	0.04
Ndufa4	-0.5	0.0019	0.04
Ndufa2	-0.6	0.0021	0.04
Ndufb9	-0.5	0.0024	0.05
Cox6a1	-0.5	0.0024	0.05
Atp5l	-0.6	0.0025	0.05

Table S2. EpoR deletion is associated with changes in the expression of mitochondrial genes.
